# Prosecution for Dental Fees

**Published:** 1875-06

**Authors:** 


### ARTICLE II.

### *Prosecution for Fees.*

BY A SCARBOROUGH, ENGLAND, DENTIST.

PEACOCK *vs.* HARRISON.—This was a curious action, in which much interest was manifested during the whole of the time it was under hearing. It was a case affecting the charge of a local dentist, in which the plaintiff, Mr. Charles James Peacock, dentist, Scarborough, claimed the sum of £4 4s. from the defendant, Mr. J. W. Harrison, timber merchant, Cottingham, near Hull, for filling one of his daughter's teeth, and operating on another tooth, the operation lasting four and a half hours, in October, 1873. The defendant's daughter is Miss Mabel Harrison, who had been a pupil in Miss Stephens' boarding-school, Denmark House, Scarborough. It was objected on the part of the defendant that the charge was excessive, and defendant had paid one guinea into court, pleading that that was fair and reasonable compensation to the plaintiff. For the plaintiff there appeared Mr. Warner Sleigh (specially retained) and Mr. Haigh, instructed by Mr. H. O. Welburn, solicitor. Mr. John Smith was counsel for the defendant, instructed by Messrs. Cornwall, Watts, and Crowther.

Mr. Sleigh opened the case in a speech of much ability and considerable length. The plaintiff was, he said, a gentleman who must be well known to the jury, for he had deservedly gained during his course of practice a very high reputation for skill, patience, knowledge, and success in his profession as a dentist. He had been educated as a dentist, and he had received his diploma from a college which existed in America, and which he (the learned counsel) was sorry to say there was nothing like in this country— it was a college for the study of dental surgery. Having received that education, having passed that college, and having got his diploma, he had been practising in England for some years. His fee was now a guinea an hour, and it was well known that that was his charge. Mr. Peacock, from his skill and experience, thought he had a right to make this charge, and it was one that had been for some time ungrudingly paid by his patients, who considered it a fair and proper charge. The defendant was a gentleman who resided in the neighborhood of Hull, and in the month of October, 1873, his daughter, Miss Mabel Harrison, was at school at Scarborough, at the seminary of Miss Stephens. She suffered there very much from toothache—several of her teeth being in decay—and on the advice of Miss Stephens she sought the permission of her parents to go and visit the plaintiff, so that one or more of her teeth which were decaying should be stopped. The requisite permission was given, and on the 13th of October she went to the plaintiff's residence to go through the operation. She was there several hours, Mr. Peacock and his assistant performing two distinct operations on her. One was the filling with gold of one of her jaw or molar teeth, whilst the other was preparing the next tooth to it, which was a dead tooth —so that it might be operated upon at a future day. The two operations were not completed under four hours and a half, and Miss Harrison made an appointment with the plaintiff that she would go on the 15th and have the second tooth further attended to. That appointment was not kept, however, and on the 24th the plaintiff got a letter sent him by Mrs. Harrison, asking how long he would take to stop her daughter's tooth, and stating that she should not send Miss Mabel a second time unless Mr. Peacock would say how much he was going to charge. It would be for the jury to say, but he (the learned counsel) contended that the fair interpretation to be put upon that letter was that Miss Harrison's parents, or at any rate Mrs. Harrison, knew that the plaintiff was in the habit of charging so much an hour; indeed he should be able to prove beyond that, that they must have known it was a guinea an hour. Well, to go back to the 15th, the appointment was not kept, but for that, though plaintiff was in the habit of making his engagements a long time in advance, (owing to the extent of his connection,) and charging a guinea if they were not kept, he had not made any claim. He claimed four guineas for his work on the 13th. The bill was sent in the ordinary way to the defendant at Christmas, 1873, but payment was not pressed, and the account stood over unsettled. They would have thought that if the defendant had then thought of contesting the charge on the ground that it was unreasonable, and under the belief that he would be justified in so doing, that when the bill was sent in he would have then said something. But no ; there was no repudiation, and the thing ran on until the summer of 1874. The plaintiff then sent, in the usual way with overdue accounts, a notice to the defendant, which intimated that he would be glad if he would pay, and it was in reply to that that he received the following commnication :—

COTTINGHAM, NEAR HULL, MAY 15TH, 1873.

Mr. C. J. Peacock—

SIR :—I expected to be in Scarboro' before this time, and to have called upon you respecting yonr charge of four guineas for stopping one tooth.

I was brought up a surgeon, and have stopped teeth and drawn hundreds, but never in my professional experience heard of such a charge

If you reduce it to one guinea I will pay it; this is 10s. 6d. more than I ever was charged. I am, yours truly,

J. W. HARRISON, M. R. C. S., L. A. C.

In the above letter, said the learned counsel, Mr. Harrison went out of his way to falsify the facts, for he here said plaintiff was charging four guineas for stopping one tooth, when the fact actually was, as he had stated, there were two operations, viz: the stopping of one tooth and the preparing of another. To the above letter Mr. Peacock replied as follows:—

SIR.—Before your daughter came to me she knew from Miss Stephens that my fee was a guinea an hour. It took two of us four hours and a half to perform the important operation, for which I have only charged. four guineas. My practice is much larger than I am able to attend to, and if I had never seen your daughter some one else would have occupied the time I devoted to her. As regards the truth of my statements I refer you to Miss Stephens. Having rendered your daughter the service of which she stood greatly in need, in the way of my profession, to the best of my ability, I now call upon you to satisfy my demand for just compensation for the time, labor, and what skill I may possess, I have given to her case. Yours obediently,

C. J. PEACOCK.

That, the learned counsel held, was a fair and gentlemanly way of putting the matter to defendant, and a way in which a fully qualified professional man who had given his time and his talents was entitled to put it. From that time Miss Harrison was removed from Scarborough, and defendant, he supposed, now imagined that he could ignore plaintiff's claim, because he took no notice of the letter just read. He (Mr. Sleigh) could not help thinking that the defendant might at any rate have decently acknowledged the receipt of the communication, but he had not thought it worth while to reply, and had left Mr. Peacock, to recover his money if he could. This was not the first time the action had been before the Recorder. When brought the first time before the Court it was withdrawn by reason of the absence of a witness, so that accounted for the lateness with which the present jury had to deal with it. Now, if the plaintiff was entitled to his four guineas he was perfectly right in fighting this case for the principle of the thing, cost what it might; in fact, now that the challenge was thrown down it would be cowardly if he did not take it up. Having a fixed scale of charges, and having brought his skill and knowledge to bear in the service of defendant, his client felt it would be an insult to himself and derogatory to his profession to accept any less. This was the principle on which his client came to the jury, and declined to accept the paltry guinea proffered by the defendant. The only matter in dispute was as to the amount charged, and he contended that it was clear from the letters read that Miss Harrison's friends knew there was a stipulated charge; and if they did not know the precise amount in the first instance, it was clear they had accepted the charge made for the first visit, because Mrs. Harrison said her daughter was not to go again without she knew what the precise charge would be for a certain operation. But he should go further than this, and he would be able to show that Miss Stephens knew quite well that a guinea per hour was Mr. Peacock's charge, and that her pupils were in the habit of paying that charge.

Mr. Smith remarked that there was no proof of contract.

Mr. Sleigh said he was anxious to try the case on its merits, as between man and man, and if it was the policy of the other side to throw every possible technical objection in the way of his client, in order to prevent him obtaining his just demands, why the jury would form their own opinion of such conduct. There were dentists and dentists, some who performed their duty with ability, and who were really and truly benefactors to their race; others there were who were quacks and charlatans, and had no more right to put an instrument into the mouth of a fellow-being than they had to do any other act which caused death through negligence or want of skill. There was, as he had regretted, no college of dentists in England, and it was a very easy thing for a man to get a brass plate up and call himself a dentist in this country; it required by law no education for the work, no diploma, no qualification. It might be, and doubtless was, that some who called themselves dentists might be able to fill up a holed tooth in a very short time—might, in fact, be able to do twenty fillings up whilst another man more competent took all the time to do one, and do it properly and effectually. The learned counsel then minutely described the operation of filling up a tooth in the manner in which he said it was necessary it should be done, pointed out that the material used, gold leaf, was very expensive, and then intimated, that in order to support the plaintiff in his declaration that the charge made was a fair and reasonable one, as well as the one understood to be his charge, he should call a number of witnesses whose testimony would be valuable, including one of the most successful and experienced dentists of the present day, Dr. Coffin, of London.

Mr. Smith remarked that he questioned whether some of the evidence spoken of would, when it came to be tendered, be admissable.

Mr. Sleigh contended it would, and observed that Mr. Smith was now beginning to feel that it was getting a little too hot. (Laughter.) He (Mr. Sleigh) thought it came with ill grace from the defendant who was contesting the claim, to try and exclude this evidence as to the competency of the plaintiff and the fairness of his charges.

The Recorder hinted that the evidence would be perfectly proper, and he further observed that if the defendant knew what the charge was going to be he should rule that no evidence as to fairness was necessrry.

Mr. Smith.—Unless the work is badly done.

The Recorder.—Certainly.

Mr. Sleigh.—Oh, that is another card my friend is going to play. (Laughter.) Till to-day it has never been suggested that the work was not properly done. However, I am perfectly willing to take it upon that ground.

He proceeded to say that Mr. Peacock was a gentleman of education, he had taken every trouble to make himself fully acquainted with the details of his profession, he had a diploma from the Dental College of Pennsylvania, and having for some years practised the profession with success in this country, he was entitled to be fairly remunerated for his skill and knowledge. Witnesses for the plaintiff were then called, and were examined by Mr. Haigh.

Miss Stephens was the first witness called. She said she kept a young ladies' school at Denmark House, Scarboro'. In October, 1873, Miss Mabel Harrison was at her school, and complained about her teeth. Witness told her she had better see a dentist, and she named Mr. Peacock, the plaintiff, advising her to write to her parents, asking them to give their assent to her seeing him. Mr. Peacock had usually attended her pupils, and she knew his fee to be a guinea an hour. It was well known amongst the pupils what that fee was, and witness told Miss Harrison that it would be a guinea an hour. Miss Harrison got a letter from her mother, and in consequence of that she went to Mr. Peacock. She went at about ten o'clock in the morning on the 13th of October, and returned a little after three o'clock. Previous to that Miss Harrison had suffered a good deal from her teeth, which were very decayed. After seeing Mr. Peacock she became quite well.

Mr. Charles James Peacock, the plaintiff, was then sworn. He said he was a graduate by examination of the Pennsylvania College of Dental Surgery, and his diploma was in Court, (produced.) He studied in America expressly for the purpose of being a dentist. He had been in practice about nine years in Scarboro', and had a very considerable connection. Witness then gave evidence regarding the correspondence alluded to by Mr. Sleigh. The fee of one guinea an hour was a fair and reasonable charge, and one which was always paid to him by all his patients. His appointments were always made in advance, and he occasionally charged a guinea if an appointment was not kept. That was not charged in this instance to the defendant because of his daughter not coming a second time. Pupils from Miss Stephens' school had come to him before, and he had been paid a guinea an hour. Witness then spoke as to the operations he performed on Miss Harrison on the 13th of October. He prepared and filled with gold a molar tooth and was about three hours at that. The other tooth had the pulp exposed and diseased, and he applied medicine to destroy the nerve and then put in a temporary filling to keep the medicine in. That operation took about an hour and a half. To do justice to the patient he could not have occupied less time than four hours and a half for both. Compared with other dentists the fee was a fair and reasonable one. He knew the fees of London dentists. He had himself paid to Dr. Coffin £60 for 19 fillings. Dr. Coffin told him that if he (plaintiff) had not been a professional man his fee would have been two guineas an hour.

By Mr. Smith.—I am 40 years of age. I was in a Manchester warehouse before studying to become a dentist, and I was at Phillips' at Manchester. The diploma is dated 1861. I had two sessions at the college. A session is four months and a half.

Mr. Smith.—How much did it cost you, may I ask?

Witness.—Oh, about £200 altogether.

Mr. Smith.—Not the diploma; what did the diploma cost you?

Witness.—Oh, some nominal matter. I forget now. I believe about twenty dollars was the cost of the parchment.

Mr. Smith.—And I suppose that would be paid in greenbacks.

Witness.—No; they had not greenbacks in America then. (Loud laughter.)

Further cross-examined by Mr. Smith. Witness said that the tooth, if it was properly stopped, ought to do for years if the brush was liberally used. I have charged 10s. 6d. for filling a tooth, but that is some time since. I used to charge from 10s. 6d. to two guineas a tooth, but in July, 1871, I raised my fees to a guinea an hour.

Dr. Hickson, of Scarboro', said he knew the plaintiff as a dentist, and knew his fee to be one guinea an hour. Had many a time sent patients of his to the plaintiff since that was his fee. Witness had himself been under his treatment. He considered him, he might as well say it at once, one of the first dentists in Europe. He did not think the fee was unreasonable.

Dr. Coffin, of Cornwall Gardens, Queen's Gate, London, and a graduate of the Baltimore College of Dental Surgery, was next called. He said that for eighteen years he had practiced in London and in the United Kingdom ; did not think the charge made by the plaintiff considering what he had done,was enough. Witness charged two guineas an hour He had seen Mr. Peacock's work, a good deal of it for thirteen years, and he thought very highly of it. There was complaint of every dentist's work, but he thought there was less complaint of Mr. Peacock's than most people. He could speak in the highest praise of him. The process of filling a tooth should be a slow one when done properly. Of course witness had not seen the operation in question, but judging from what he heard it was not too long.

Dr. Horne, of Scarboro', said he had frequently been under plaintiff's hands. His fee was one guinea per hour. He had a very good opinion of him. For the skillful services he rendered he thought the charge most moderate.

Dr. Teale, of Scarboro', said that plaintiff had stopped two teeth of his. From his experience of the plaintiff, both as a patient himself, and from what he knew of patients of his who had been under him, he had a very high opinion of him. He had seen a good deal of good work of his, and no bad. His fee of a guinea an hour had for some time been well known by witnesses patients.

This concluded the plaintiff's case.

Mr. Smith addressed the jury very briefly for the defense. He began by depreciating the value of plaintiff's diploma as one which could very easily and cheaply be got, and in regard to his mode of charging said that would enable him him to dawdle over his work as long as he thought proper. He suggested that it had been done in this instance; but further than that, Miss Harrison would be called, and she would show that even with that the work was not done properly, for part of the stuffing had since come out. She had been subpœnaed by the plaintiff, but they had singularly enough not thought fit to call her. As to the evidence given by the doctors whom the plaintiff had called, he (Mr. Smith) did not wish to impugn their evidence, but they were all more or less interested in keeping up high fees. (Laughter.) He would only call Mr. Harrison, a dentist of London and Hull, who would say that a guinea would be a reasonable charge, and after that the case would be in their hands.

Miss Mabel Harrison, the defendant's daughter, stated that she went at ten o'clock on the morning in question to see Mr. Peacock. She had to wait about half an hour, and whilst she remained there the plaintiff left twice, being absent altogether a quarter of an hour. She got home at half-past one or two. Last Christmas a portion of the stuffing came out.

To Mr. Sleigh.—I have had four or five teeth performed on. Since I was at Mr. Peacock's I have been to Mr. Harrison's, at Hull, who comes from London.

Mr. R. H. Harrison (no relative of the defendant's) said he was a dentist in London and Hull.

Mr. Smith.—How long have you been one?

Witness.—I have been one ever since I was anything.

Mr. Sleigh.—What a precocious infant he must have been. (Laughter.)

Whereupon one of the legal gentlemen observed that it was very natural witness should have begun life by "cutting his teeth," an acute remark that did not seem to catch the Recorder's ear.

Witness continued.—I consider one guinea, or at the most two, an ample fee for a provincial dentist.

Witness added in answer to Mr. Smith, that he was a Licentiate in Dentistry of the Royal College of England—not of America.

Mr. Sleigh.—Don't you know that the Americans are the most proficient dentists in the world?

Witness.—I know that they claim to be.

Mr. Sleigh.—Are they not recognized as such?

Witness.—I should be very sorry to think so.

Mr. Sleigh.—We don't want to know anything about your sorrow. I ask you whether they are not considered such?

Witness.—I cannot tell what other medical men think.

Mr. Sleigh.—Do you deny that in medical and scientific circles the Americans are considered the best dentists in the world?

Witness.—I don't deny it, and I don't admit it.

The Recorder having briefly summed up the evidence, the jury retired, and after a short absence returned into Court with a verdict for the full amount.

Mr. Sleigh applied for costs.

The Recorder certified that it was a fit case to be brought into that Court, and he therefore should order that the costs should be paid according to the usual scale of the Court.

